# The impact of informant-related characteristics including sex/gender on assessment of Alzheimer's disease symptoms and severity

**DOI:** 10.3389/fgwh.2024.1326881

**Published:** 2024-03-28

**Authors:** E. Abken, M. T. Ferretti, Laura Castro-Aldrete, A. Santuccione Chadha, M. C. Tartaglia

**Affiliations:** ^1^ Women’s Brain Project, Guntershausen bei Aadorf, Switzerland; ^2^ London School of Hygiene and Tropical Medicine, London, United Kingdom; ^3^ Center for Alzheimer Studies, Karolinska Institute, Stockholm, Sweden; ^4^ Tanz Centre for Research in Neurodegenerative Disease, University of Toronto, Toronto, ON, Canada; ^5^ Memory Clinic, Krembil Brain Institute, University Health Network, Toronto, ON, Canada

**Keywords:** Alzheimer's disease, caregive, sex, gender, Clinical Dementia Rating (CDR), clinical trial endpoints

Alzheimer's disease (AD) is a debilitating neurological disorder affecting millions of people worldwide. Early and accurate diagnosis of AD is crucial for accessing treatments (including clinical trials), planning for the future, and obtaining necessary services. Unfortunately, AD and other neurodegenerative diseases associated with cognitive impairment or behavioral changes, are frequently misdiagnosed or diagnosed at later stages. Experts recommend a multifaceted approach that integrates performance, informant, and self-report data to properly assess patients with neurodegenerative diseases (1). This approach allows clinicians to assess the presence and severity of cognitive disorders, establish a differential diagnosis, and formulate an effective treatment plan. Caregivers play a pivotal role in recognizing early signs of the disease, such as memory loss and behavioral changes. They provide valuable insights to healthcare providers, assisting in diagnosis and treatment (2). In contrast to conditions where self-reporting is standard, caregiver input is instrumental in AD assessments, especially in clinical trials where disease severity is a critical criterion and endpoint.

Integral to an accurate ascertainment of a patient's severity of disease, which is focused on functional abilities, is a dementia rating scale. In the Clinical Dementia Rating Scale (CDR), a semi-structured interview format is used to collect detailed information from an informant regarding the patient's ability to function in various domains (3). The CDR (either the global score, from 0 (no impairment) to 3 (severe dementia), or the sum of boxes, CDR-SB) is thought to reflect the impact of neurodegeneration on everyday global function based on six cognitive and behavioral domains: memory, orientation, judgment and problem solving, community affairs, home and hobbies, and personal care. Currently, this is the most widely used scale for assessing severity/staging of AD (4, 5). The CDR-SB is commonly used as the primary endpoint in clinical trials of AD, in addition to several cognitive measures.

Given the importance of the CDR, it is imperative to consider the informant's characteristics that may influence scoring. Prior research highlights the significance of caregiver input in diagnosing and classifying AD and other neurodegenerative diseases (2). Psychosocial factors, including cultural background (6), ethnicity (7–9), and sex/gender (10) can influence the diagnosis of neurodegenerative diseases (11). However, it's reasonable to hypothesize that additional informant characteristics such as their relationship with the patient, time spent with the patient, their gender/sex, and cultural and socioeconomic factors may also impact diagnosis and severity scores. It's essential to consider these factors when analyzing caregiver reports, as they can introduce biases in patient diagnosis and staging, particularly since severity assessments are primary endpoints in clinical trials. Examples of such factors include:

1. The informant's *relationship with the patient* can significantly affect their perception of the patient's health status, potentially influencing severity scores. One study has shown that wives tend to be more optimistic about the progression of the disease compared to husbands (12), potentially affecting patient staging in informant-based assessments. The amount of time informants spend with the patient may also impact severity assessments. Caregivers who are closely involved with the patient might have a better understanding of their condition, but their assessments may be colored by factors such as caregiver stress or habituation to symptoms (13, 14). Alternatively, caregivers who are not living with the patient, such as children or friends, may not accurately assess the patient's functioning, especially in the early stages of the disease. Additionally, the nature of the relationship between the informant and the patient, whether a spouse, child, or in-law, may affect assessment accuracy. They may underestimate or overestimate their function based on their limited knowledge of previous functional ability in certain areas such as finances or household management.
2. *The sex/gender* of the informant can also influence dementia assessments in significant ways. Gender influences the awareness and likelihood of caregivers to report symptoms about patients with AD. Women often have a key role in detecting and reporting symptoms in their partners due to their close relationships and caregiving responsibilities. They tend to be more perceptive of subtle changes in cognition, behavior, and daily functioning, leading to early detection and intervention for AD (15). Previous research has shown that female caregivers are more likely to notice and report symptoms of depression in patients (16), which may be a risk factor for dementia as well as a prodromal symptom (17–19). Given that approximately 67% of family caregivers are women, with 80%–90% of them being adult children [National Alliance for Caregiving and AARP (20)], caregiver gender should be considered when interpreting reports. Additionally, gender stereotypes may unintentionally influence the evaluation scale used to assess AD symptoms, affecting the interpretation of caregiver reports.
3. *Cultural and socioeconomic characteristics* can influence how caregivers assess AD symptoms and severity (21, 22). These factors can include language barriers, cultural beliefs, and financial constraints. An investigation into behavioral and psychological symptoms in AD patients underscored the significance of caregiver attributes, including their education level, age, gender, co-residence with the patient, and time spent caregiving, in relation to the severity ratings on the Neuropsychiatric Inventory (23).

In summary, informants and caregivers are instrumental in identifying and assessing cognitive and behavioral deficits in AD and other neurodegenerative diseases, owing to their close connection with the patient. Informant attributes, such as gender, relationship to the patient, the amount of time spent with the patient, and cultural and socioeconomic factors, may impact the evaluation process. Given the importance of assessments in recruiting patients for clinical trials and tracking disease progression, understanding these factors is crucial. Incorporating caregiver characteristics allows clinicians to account for potential biases in reports, improving the accuracy of dementia staging and response to therapy.

There is an urgent need for the development of precise assessment tools that address the limitations of current scales. By advancing scale development, including caregiver characteristics, incorporating biomarkers and digital technologies, we can obtain objective measures, enhance early detection, and enable personalized interventions. This comprehensive approach will significantly improve patient care and outcomes in neurodegenerative disease.

## References

[1] McKhann GM Knopman DS Chertkow H Hyman BT Jack CR Kawas CH The diagnosis of dementia due to Alzheimer’s disease: recommendations from the national institute on aging-Alzheimer’s association workgroups on diagnostic guidelines for Alzheimer’s disease. Alzheimers Dement. (2011) 7(3):263–9. 10.1016/j.jalz.2011.03.005 21514250 PMC3312024

[2] Roberts JL Clare L Woods RT. Subjective memory complaints and awareness of memory functioning in mild cognitive impairment: a systematic review. Dement Geriatr Cogn Disord. (2009) 28(2):95–109. 10.1159/000234911 19684399

[3] Moelter ST Glenn MA Xie SX Chittams J Clark CM Watson M The dementia severity rating scale predicts clinical dementia rating sum of boxes scores. Alzheimer Dis Assoc Disord. (2015) 29(2):158–60. 10.1097/WAD.0000000000000031 24770371 PMC4208973

[4] Aisen PS Gauthier S Ferris SH Saumier D Haine D Garceau D Tramiprosate in mild-to-moderate Alzheimer’s disease—a randomized, double-blind, placebo-controlled, multi-centre study (the alphase study). Arch Med Sci. (2011) 1:102–11. 10.5114/aoms.2011.20612 PMC3258678 22291741

[5] Balsis S Benge JF Lowe DA Geraci L Doody RS. How do scores on the ADAS-Cog, MMSE, and CDR-SOB correspond? Clin Neuropsychol. (2015) 29(7):1002–9. 10.1080/13854046.2015.1119312 26617181

[6] Wood JB Parham IA. Coping with perceived burden: ethnic and cultural issues in Alzheimer’s family caregiving. J Appl Gerontol. (1990) 9(3):325–39. 10.1177/073346489000900307

[7] Tang MX. The APOE-∊4 allele and the risk of Alzheimer disease among African Americans, whites, and hispanics. JAMA. (1998) 279(10):751. 10.1001/jama.279.10.751 9508150

[8] Mehta KM Yaffe K Perez-Stable EJ Stewart A Barnes D Kurland BF Race/ethnic differences in AD survival in US Alzheimer’s disease centers. Neurology. (2008) 70(14):1163–70. 10.1212/01.wnl.0000285287.99923.3c 18003939 PMC2830859

[9] Santamaria-Garcia H Sainz-Ballesteros A Hernandez H Moguilner S Maito M Ochoa-Rosales C Factors associated with healthy aging in Latin American populations. Nat Med. (2023) 29(9):2248–58. 10.1038/s41591-023-02495-1 37563242 PMC10504086

[10] Salwierz P Davenport C Sumra V Iulita MF Ferretti MT Tartaglia MC. Sex and gender differences in dementia. In: Moro E, Arabia G, Tartaglia MC, Ferretti MT, editors. International Review of Neurobiology. Elsevier (2022). p. 179–233. Available online at: https://linkinghub.elsevier.com/retrieve/pii/S0074774222000733 (cited 2023 October 19, 2023). 10.1016/bs.irn.2022.07.002 36038204

[11] Bunn F Goodman C Sworn K Rait G Brayne C Robinson L Psychosocial factors that shape patient and carer experiences of dementia diagnosis and treatment: a systematic review of qualitative studies. PLoS Med. (2012) 9(10):e1001331. 10.1371/journal.pmed.1001331 23118618 PMC3484131

[12] Kokorelias KM Naglie G Gignac MA Rittenberg N Cameron JI. A qualitative exploration of how gender and relationship shape family caregivers’ experiences across the Alzheimer’s disease trajectory. Dementia. (2021) 20(8):2851–66. 10.1177/14713012211019502 33998323 PMC8678646

[13] Ostergren JE Heeringa SG Leon CFMD Connell CM Roberts JS. The influence of psychosocial and cognitive factors on perceived threat of Alzheimer’s disease. Am J Alzheimers Dis Dementiasr. (2017) 32(5):289–99. 10.1177/1533317517714552 PMC5886712 28605999

[14] Pratt R Wilkinson H. A psychosocial model of understanding the experience of receiving a diagnosis of dementia. Dementia. (2003) 2(2):181–99. 10.1177/1471301203002002004

[15] Roberto KA Jarrott SE. Family caregivers of older adults: a life span perspective. Fam Relat. (2008) 57(1):100–11. 10.1111/j.1741-3729.2007.00486.x

[16] Yee JL Schulz R. Gender differences in psychiatric morbidity among family caregivers. Gerontologist. (2000) 40(2):147–64. 10.1093/geront/40.2.147 10820918

[17] Steenland K Karnes C Seals R Carnevale C Hermida A Levey A. Late-life depression as a risk factor for mild cognitive impairment or Alzheimer’s disease in 30 US Alzheimer’s disease centers. J Alzheimers Dis. (2012) 31(2):265–75. 10.3233/JAD-2012-111922 22543846 PMC3729228

[18] Ritchie K Carrière I Berr C Amieva H Dartigues JF Ancelin ML The clinical picture of Alzheimer’s disease in the decade before diagnosis: clinical and biomarker trajectories. J Clin Psychiatry. (2016) 77(03):e305–11. 10.4088/JCP.15m09989 26891108

[19] Ownby RL Crocco E Acevedo A John V Loewenstein D. Depression and risk for Alzheimer disease: systematic review, meta-analysis, and metaregression analysis. Arch Gen Psychiatry. (2006) 63(5):530. 10.1001/archpsyc.63.5.530 16651510 PMC3530614

[20] National Alliance for Caregiving, AARP. Caregiving in the U.S. 2020. Washington, DC: AARP (2020). Available online at: https://www.aarp.org/ppi/info-2020/caregiving-in-the-united-states.html (cited October 19, 2023).

[21] Brodaty H Donkin M. Family caregivers of people with dementia. Dialogues Clin Neurosci. (2009) 11(2):217–28. 10.31887/DCNS.2009.11.2/hbrodaty 19585957 PMC3181916

[22] Ibáñez A Pina-Escudero SD Possin KL Quiroz YT Peres FA Slachevsky A Dementia caregiving across Latin America and the Caribbean and brain health diplomacy. Lancet Healthy Longev. (2021) 2(4):e222–31. 10.1016/S2666-7568(21)00031-3 34790905 PMC8594860

[23] Sink KM Covinsky KE Barnes DE Newcomer RJ Yaffe K. Caregiver characteristics are associated with neuropsychiatric symptoms of dementia. J Am Geriatr Soc. (2006) 54(5):796–803. 10.1111/j.1532-5415.2006.00697.x 16696746

